# Engineering the Water/Salt Sorption Selectivity of Polymers for Desalination Applications

**DOI:** 10.1021/acsmacrolett.5c00449

**Published:** 2025-10-11

**Authors:** Sean M. Bannon, Rachel L. Fetter, Natasha E. D’Cunha, Geoffrey M. Geise

**Affiliations:** Department of Chemical Engineering, 2358 University of Virginia, 385 McCormick Road, Charlottesville, Virginia 22903, United States

## Abstract

In this paper, we report two synthetic strategies to engineer the water/salt sorption selectivity of polymers: tethering polar functional groups to the polymer backbone and increasing the degree of cross-linking. For the first strategy, we found that at a given water content, the dielectric constant of hydrated methacrylate-based polymers functionalized with hydroxyethyl (i.e., two carbon) side chains (XL – p­(HEMA-*co*-GMA) is less than that of hydrated methacrylate-based polymers with hydroxypropyl (i.e., three carbon) side chains (XL – p­(HPMA-*co*-GMA), which contributes to suppressing salt sorption to increase the water/salt sorption selectivity. For the second strategy, we found that forming densely cross-linked polymers that contained only dimethacrylate-based monomers (XLPEGDMA) relative to less densely cross-linked copolymers containing both methacrylate- and dimethacrylate-based comonomers (XL – p­(HEMA)) reduced the network mesh size at a given water content, which also suppressed salt sorption and increased the water/salt sorption selectivity. These structure–property results inform the design of advanced materials for desalination membrane applications.

## Graphical abstract

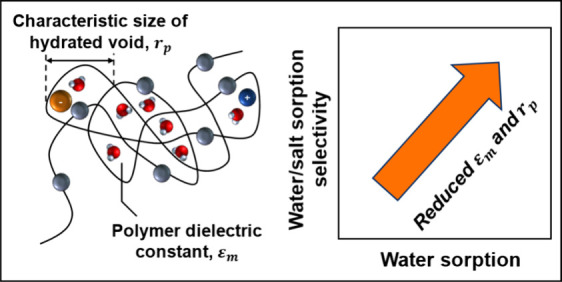

Polymer-based membranes are used in desalination processes that selectively separate salt (i.e., ions) from water to address global water shortage issues.^1–5^ Of these separation processes, reverse osmosis (RO) is most widely used on the industrial scale to desalinate water.^1,3^ In RO, desalination separation is achieved if the fluxes of water and salt through the membrane are different, and from a materials design perspective, these fluxes can be tuned independently by controlling the intrinsic mass transport properties of the polymer that is used as the selective layer in state-of-the-art membrane materials.^4–8^

One strategy to increase the water flux through a polymer membrane relative to the salt flux is to increase the water/salt sorption (or partitioning) selectivity of the polymer.^9–12^ This sorption selectivity, *K*_w_/*K*_s_, is the ratio of the water and salt sorption coefficients, which are defined as the solution-concentration normalized concentrations of water and salt in the polymer, respectively.^4,13,14^ The water/salt permeability selectivity is the product of this sorption selectivity and the ratio of the water and salt diffusion coefficients.^8^ When the sorption selectivity increases, water sorption is enriched in the polymer relative to salt sorption (i.e., salt is increasingly rejected from partitioning into the polymer) and water/salt permeability selectivity is expected to increase due to the proportionality between sorption and permeability properties.

To develop strategies to engineer the water/salt sorption selectivity of uncharged hydrophilic polymers, the desalination literature contains reports of polymer synthesis and water/salt sorption selectivity characterization, and these studies often use aqueous NaCl, which is typically the most abundant salt in feeds to desalination processes.^4,6,10,15–18^ In these reports, the water/salt sorption selectivity varies, by approximately an order of magnitude, in the range 1 to 10, which provides a benchmark for the range of sorption selectivity property variation in the types of materials relevant to this report. In one study, on materials like those that are the subject of this report, manipulation of the hydroxyl group configuration in a series of hydroxyl-containing methacrylate-based polymers (with water volume fractions of 0.23) led to an increase in the water/salt sorption selectivity from 1.9 to 2.9, and this approximately 50% increase occurred as the hydroxyl group distribution was varied from a vicinal diol-rich to single hydroxyl-rich configuration.^10^ While these previous studies are useful to understand the relationship between polymer structure and polymer water/salt sorption selectivity properties, opportunities still exist to clarify how synthetic variables (e.g., the ratios of comonomers used as reagents during polymer synthesis) can be modified to control rationally the water/salt sorption selectivity properties of materials for desalination applications.

The processes of water and salt sorption from an aqueous electrolyte into a hydrated polymer are governed by thermodynamic interactions between water molecules, ions, and the solvated polymer.^19–22^ Continuum-level thermodynamic models that describe these interactions, which are often electrostatic in nature, can be used to describe accurately these interactions in hydrated polymers and inform how polymer physical properties relate to their water/salt sorption selectivity properties.^19–22^ For example, our recent application of the Freger–Born model^22^ (which is based on Freger’s pore model for hydrated polymers^23^ and Born’s classic model of ion solvation^24^) suggests that, assuming the water volume fraction is approximately equal to the water sorption coefficient, the water salt sorption selectivity scales according to^22^

1 $$\frac{K_{w}}{K_{s}}∼\exp\left[C_{h}\frac{1}{r_{p}}\left(\frac{1}{\varepsilon_{m}}-\frac{1}{\varepsilon_{s}}\right)\right]$$

where *C*_h_ is a hydration constant related to the salt valence and solution temperature, *r*_p_ is the characteristic size of the interstitial hydrated void space between polymer chains (which can be approximated using the network mesh size for cross-linked polymers), and ε_m_ is the hydrated polymer dielectric constant. The Freger–Born model suggests that any modification of the polymer structure that influences the characteristic hydrated void space and/or the hydrated polymer dielectric constant should influence the water salt sorption selectivity of the polymer. These theoretical insights highlight opportunities to leverage emerging synthetic strategies, such as those that allow for precise control over polymer morphology and functional group placement, to develop molecular engineering strategies that improve the water/salt sorption selectivity of polymer-based desalination membranes.

The goal of this communication is to report strategies to engineer the water/salt sorption selectivity of hydrated polymers via control of polymer chemistry at the synthetic level (reporting strategies that engineer the diffusion selectivity of these polymers is outside of the scope of this study). Specifically, we report that distributing hydroxyethyl functionality throughout the polymer, relative to hydroxypropyl functionality, suppresses the dielectric constant and improves the water/salt sorption selectivity of hydroxyethyl methacrylate/glycidyl methacrylate-based copolymers relative to that of hydroxypropyl methacrylate/glycidyl methacrylate-based copolymers. Second, we report that increasing the degree of cross-linking in polymers to reduce the characteristic network mesh size increases the water/salt sorption selectivity of densely cross-linked poly­(ethylene glycol) dimethacrylate relative to less densely cross-linked and linear poly­(hydroxyethyl methacrylate)-based polymers. These results, which inform the sorption selectivity properties of the polymers using independent measurements of polymer structural properties (e.g., the mesh size and dielectric constant), are qualitatively consistent with the Freger–Born model predictions. These results are useful to guide engineering strategies that inform the rational design of membrane materials for desalination processes.

The molecular polarizability of water in a hydrated polymer, which is related to the polymer dielectric constant, can be engineered by controlling interactions between water molecules and the solvated polymer.^11,25–27^ As a result, the polymer dielectric constant and, thus, within the context of the Freger–Born model, the water/salt sorption selectivity (eq 1), can be engineered by distributing hydrophilic functionalities that influence the molecular motions of water throughout the polymer matrix. To systematically vary these water/polymer interactions in hydrated polymers, we cross-linked two series of glycidyl methacrylate-based copolymers that contained either hydroxyethyl methacrylate (HEMA) or hydroxypropyl methacrylate and are referred to herein as XL – p­(HEMA-*co*-GMA) and XL – p­(HPMA-*co*-GMA), respectively (Figure 1A).

**Figure 1:**
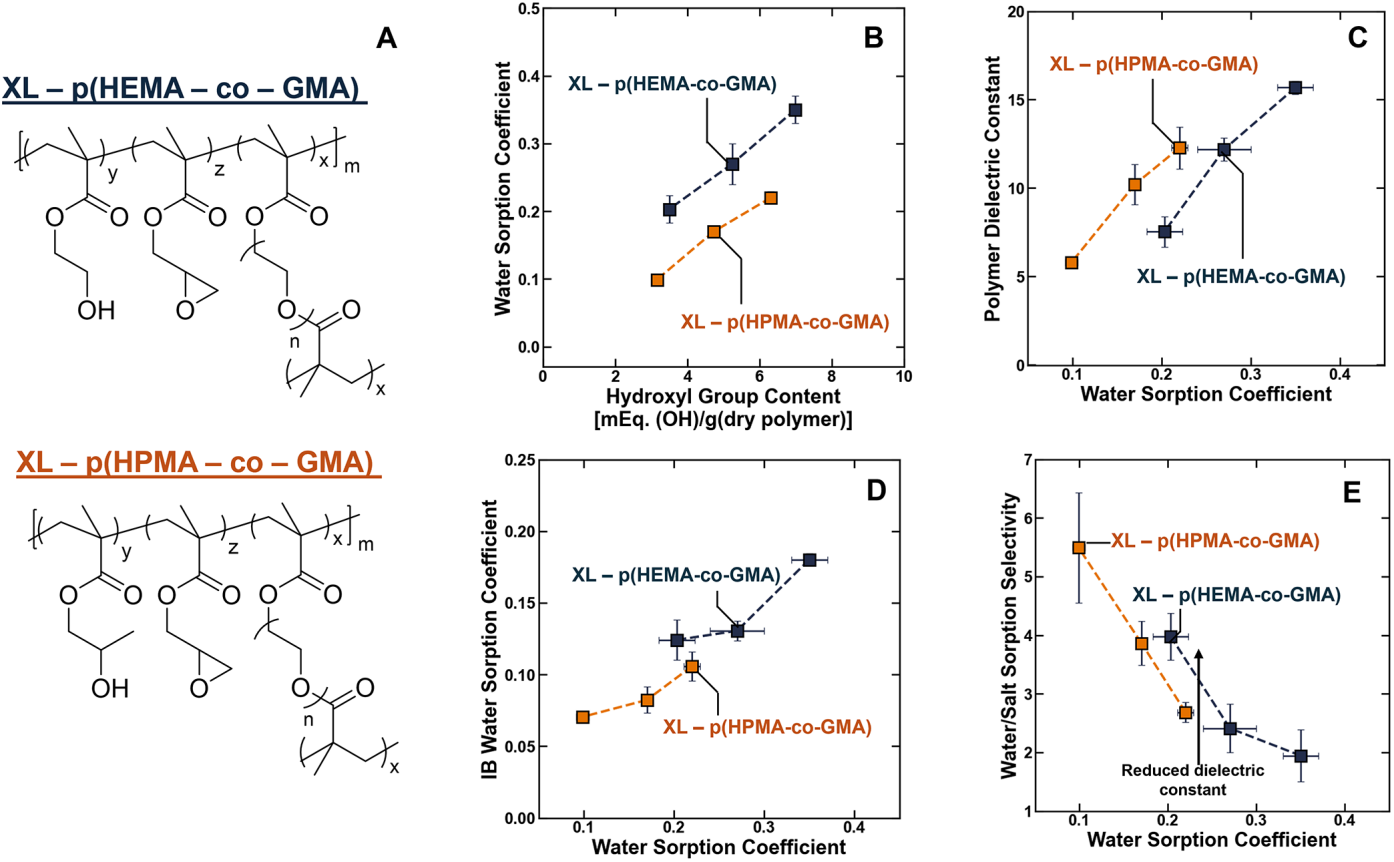
(A) Chemical structures of XL – p­(HEMA-*co*-GMA) and XL – p­(HPMA-*co*-GMA). The (B) water sorption coefficients of XL – pHEMA-*co*-GMA (black square) and XL – p­(HPMA-*co*-GMA) (orange square) plotted as a function of the hydroxyl group content (which is related to y in the chemical structure of the polymers), and the (C) dielectric constant, (D) irrotationally bound (IB) water sorption coefficient, and (E) water/salt sorption selectivity of the polymers after equilibration in 1 M NaCl plotted as a function of the total water content (i.e., the water sorption coefficient). The dashed lines are drawn to guide the eye, and the arrow is drawn to represent the influence of reduced dielectric constant on the sorption selectivity independently of the water content. The standard deviation was calculated using the mean of three measurements.

The XL – p­(HEMA-*co*-GMA) and XL – p­(HPMA-*co*-GMA) polymers were synthesized over a range of HEMA/GMA or HPMA/GMA comonomer ratios such that the resulting hydrated polymer films could be compared at similar water content, and after synthesizing the polymers, we characterized the water/salt sorption selectivity, dielectric constant, state of water, and network mesh size properties of the polymers. Note that the total water content (i.e., the water sorption coefficient) can influence the sorption selectivity properties of the hydrated polymers, so in the subsequent discussion, all materials are compared at equivalent water content such that differences in their transport properties can be ascribed directly to differences in the polymer structure.^25,28^ For brevity, the experimental details regarding these measurements are included in the Supporting Information (Section S1).

In both the XL – p­(HEMA-*co*-GMA) and XL – p­(HPMA-*co*-GMA) materials, the water sorption coefficients increased as the number density of hydroxyl groups in the polymer increased (Figure 1B). However, the water sorption coefficient of XL – p­(HEMA-*co*-GMA) was larger than that of the XL – p­(HPMA-*co*-GMA) polymers at comparable hydroxyl contents (Figure 1B). These results suggest that in the hydrated polymers, the hydroxyl groups tethered to the shorter hydrocarbon chain have increasingly favorable interactions with water molecules that promote water sorption (i.e., these groups might be considered more hydrophilic) relative to hydroxyl groups tethered to larger hydrocarbon chains. These results are generally consistent with observations made in small molecule solutions where the polarity (and thus the hydrophilicity) of an alcohol is reduced as the length of the hydrocarbon chain increases (e.g., the dipole moment of ethanol is 1.69 D and the dielectric constant of propanol is generally taken to be 1.63 ± 0.05 D).^29^

At a given water content, the dielectric constant of the XL – p­(HEMA-*co*-GMA) polymers was reduced relative to that of the XL – p­(HPMA-*co*-GMA) polymers (Figure 1C). This result is surprising because in solutions, the dielectric constant generally increases as the polarity of the molecules in the solution increases (e.g., ethanol has a dielectric constant of 25 at 20 °C, which is larger than that of propanol, which is approximately 21 at 20 °C).^30^ The dielectric constant is effectively a proportionality constant that describes the polarizability of a medium in the presence of an electric field,^26^ so in solutions, these observations generally correspond to a physical picture where, as the number (and polarity) of dipoles increases, molecules in the solution can polarize (i.e., reorient) in the presence of an applied electromagnetic field to a greater extent.

In hydrated polymers, however, this physical picture may be different. The polar functionalities tethered to the polymer backbone, relative to polar molecules in a solution, likely have more restricted rotational (i.e., dipolar) motions such that they cannot polarize to the same extent as the analogous small molecules in the corresponding liquid. For example, liquid acrylonitrile (i.e., a small molecule/monomer) has a dielectric constant of approximately 38,^31^ whereas the dielectric constant of polyacrylonitrile is reduced to approximately 5.^4^ Under these circumstances, the polar functionalities tethered to the polymer backbone may induce their own electromagnetic field that causes water molecules to orient (i.e., polarize) with the field induced by the functionality and inhibit their ability to polarize in the presence of an externally applied electromagnetic field. This structuring of water, or the formation of so-called irrotationally bound water, may contribute to reducing the average molecular polarizability of water in the polymer in a manner that reduces the dielectric constant. If the dipolar motions of these irrotationally bound water molecules are sufficiently restricted by interactions with the polymer such that they cannot solvate ions (i.e., polarize in the electric field induced by a dissociated ionic charge), salt sorption in the polymer would be inhibited by nonfavorable ion solvation interactions in the polymer relative to the external solution.

The concentration of irrotationally bound water molecules (i.e., the concentration of water in the polymer that is unable to polarize in the presence of an electromagnetic field) is larger in the hydroxyethyl-containing polymers relative to the hydroxypropyl-containing polymers at equivalent water content (Figure 1D). These results support the hypothesis that polar functionalities increase the extent that water molecules are bound (i.e., cannot polarize) in the solvated polymer. Corresponding to these observations, XL – p­(HEMA-*co*-GMA) has a larger water/salt sorption selectivity relative to XL – p­(HPMA-*co*-GMA) at comparable water content (Figure 1E). For example, at a water content of approximately 0.2., the water/salt sorption selectivity increases from 2.7 ± 0.2 in the HPMA-containing polymers to 4.0 ± 0.4 in the HEMA containing polymers. This change represents approximately a 50% increase, and statistical assessment using a *t* test to compare the XL – p­(HEMA-*co*-GMA) and XL – p­(HPMA-*co*-GMA) data at this water content yielded *t* = 5.03, which supports the statistically significant difference between the sorption selectivity properties of these two materials. Within the context of the Freger-Born model, this result is expected given the observation that hydroxyethyl functionalities suppress the dielectric constant of the polymers to a greater extent than the hydroxypropyl functionalities at a given water content (note that the characteristic hydrated void space of the polymers is effectively equal at a given water content; Figure S1). These results suggest that tethering increasingly polar functionalities to the hydrated polymer backbone to increase the concentration of irrotationally bound water (i.e., water that cannot solvate ions) improves the water/salt sorption selectivity of hydrated polymers for desalination applications.

A second technique to control the water/salt sorption selectivity of hydrated polymers is to engineer the characteristic size of the interstitial void space between the polymer chains. Previously, we demonstrated that in cross-linked polymers the characteristic network mesh size (i.e., the distance between cross-links in the network) serves as a reasonable proxy for the hydrated void space. Therefore, the characteristic hydrated void space may be engineered by controlling the amount of cross-linker used to prepare polymer networks.

Generally, the network mesh size of a polymer increases as the polymer swells in the presence of a diluent (i.e., the network mesh size scales according to the polymer water content in hydrated polymers).^32^ Therefore, to prepare materials with different network mesh sizes at equivalent water content, we prepared two series of cross-linked hydrogels: poly­(hydroxyethyl methacrylate) cross-linked with poly­(ethylene glycol) dimethacrylate, referred to as XL – p­(HEMA), and cross-linked poly­(ethylene glycol) dimethacrylate, referred to as XLPEGDMA (Figure 2A). In the XL – p­(HEMA) films, the amount of the cross-linker (i.e., PEGDMA) incorporated in the prepolymerization mixture was varied to ultimately control the network mesh size and polymer water content, and generally, as the amount of cross-linker used to prepare the polymers increased, the water sorption coefficient of the polymers decreased (Figure 2B). In the XLPEGDMA polymers, the prepolymerization water content and molecular weight of the poly­(ethylene glycol) repeat unit was varied to obtain films over a range of water content and mesh size (Figure S2), as reported previously,^33^ that could be compared to the XL – p­(HEMA) materials at equivalent water content (Figure 2B). These polymers were subject to the same characterization as the materials discussed previously, and again, for the sake of brevity, the experimental details regarding these measurements are included in the Supporting Information.

**Figure 2:**
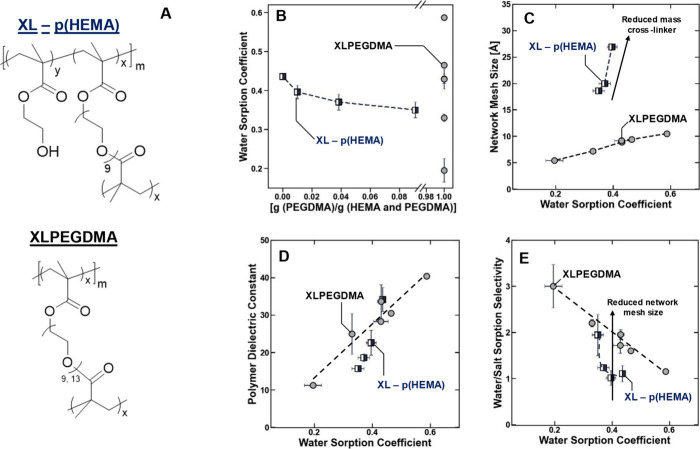
(A) Chemical structures XL – p­(HEMA) and XLPEGDMA. The (B) water sorption coefficients of XL – p­(HEMA) (black square) and XLPEGDMA (gray circle) plotted as a function of the degree of cross-linking (which is equal to *x*/(*x* + *y*) in the chemical structure, where *x* and *y* are in mass units), and the (C) network mesh size, (D) polymer dielectric constant, and (E) water/salt sorption selectivity of the polymers after equilibration in 1 M NaCl plotted as a function of the total water content (i.e., the water sorption coefficient). The dashed lines are drawn to guide the eye, and the arrow is drawn to represent the influence of the reduced network mesh size on the sorption selectivity independently of the water content. The standard deviation was calculated using the mean of three measurements.

As the amount of PEGDMA was reduced, the network mesh size of XL – p­(HEMA) increased (note that for the linear p­(HEMA) polymers (i.e., *x* = 0 (Figure 2A)), the network mesh size is not defined) (Figure 2C). For the XLPEGDMA polymers, where the water content was varied through the prepolymerization water content and molecular weight of the cross-linker (Figure S2), the network mesh size increased with increasing water content, although to a lesser extent than the XL – p­(HEMA) films where the water content was varied by changing the degree of cross-linking (Figure 2C). The network mesh size of the XLPEGDMA films was smaller than that of the XL – p­(HEMA) films, which can be explained given the significantly higher cross-linking degree of XLPEGDMA relative to XL – p­(HEMA) (in other words, incorporating linear p­(HEMA) in the network dilutes significantly the cross-links in XL – p­(HEMA) relative to XLPEGDMA) (c.f. Figure 2B and 2C)). These results are generally consistent with previous observations of the network mesh size in cross-linked networks.^33–36^

The dielectric constants of most of the XL – p­(HEMA) and XLPEGDMA polymers were statistically equal at equivalent water contents (Figure 2D). As a result, within the context of the Freger–Born model, the differences in the water/salt sorption selectivity of XLPEGDMA and XL – p­(HEMA) can be explained by using the network mesh size (eq 1). Therefore, the observation that XLPEGDMA generally exhibits a higher sorption selectivity relative to XL – p­(HEMA) (Figure 2E) is expected given that the network mesh size (i.e., the characteristic hydrated void space) of XL – p­(HEMA) is larger than that of XLPEGDMA. It is noteworthy that for the XL – p­(HEMA) polymer with the lowest water content (i.e., the highest degree of cross-linking) the water/salt sorption selectivity is effectively equal to that of the XLPEGDMA films, which may be consistent with a physical picture where the influence of the increased network mesh size on the water/salt sorption selectivity properties of the XL – p­(HEMA) relative to the XLPEGDMA is offset by a reduction in its dielectric constant (c.f. Figure 2C–E). In the Freger–Born model, these observations are consistent with a physical picture where increasing the degree of cross-linking (i.e., reducing the network mesh size) confines ions near the nonpolar polymer backbone, which contributes to increasing the extent of nonfavorable ion solvation interactions in the polymer matrix that inhibit the salt partitioning process.^22^ These results suggest that engineering the degree of cross-linking of polymer networks to control the characteristic hydrated void space can be an effective strategy to engineer the water-salt sorption selectivity of polymers for desalination applications.

In summary, the water/salt sorption selectivity properties of hydrated polymer membrane materials for desalination applications can be engineered by controlling the polymer structure. These engineering strategies can be rationally informed using fundamental theory, and using insight obtained from a continuum-level thermodynamic model referred to as the Freger–Born model, we demonstrated that tethering increasingly polar functionalities (e.g., hydroxyethyl functionalities relative to hydroxypropyl functionalities) to the polymer backbone and increasing the degree of cross-linking of polymer networks (i.e., reducing the characteristic network mesh size) improve the water/salt sorption selectivity of hydrated polymer membranes. These results are useful to guide engineering strategies for polymers used as separation devices in desalination applications, which may aid in the development of next-generation membrane materials.

## Supplementary Material



## Notes

Detailed materials and methods (PDF)
