# Supplementary material for: Engineering the Water/Salt Sorption Selectivity of Polymers for Desalination Applications
Source: ACS Macro Lett. 2025 Oct 11;14(10):1609–14. doi: 10.1021/acsmacrolett.5c00449 (PMC12548335; doi:10.1021/acsmacrolett.5c00449)
Supplement: Supplementary file 1 [file mz5c00449_si_001.pdf]

Engineering the water/salt sorption selectivity of polymers for desalination applications

*Sean M. Bannon, Rachel L. Fetter, Natasha E. D'Cunha, and Geoffrey M. Geise**

To whom correspondence should be addressed: geise@virginia.edu

Department of Chemical Engineering, University of Virginia, 385 McCormick Road,

Charlottesville, Virginia 22903

Supporting Information

Section S1. Materials

All the polymers prepared in this study were synthesized using previously reported free radical initiated polymerization procedures.[1,2] Briefly, pre-polymerization solutions containing the co-monomers, cross-linker, a free-radical initiator (which was 1-hydrocyclohexyl phenyl ketone for all materials prepared in this study), and for the XLPEGDMA polymers, water as a diluent, were mixed until homogonous. The mass percent of the initiator incorporated in the pre-polymerization solutions was either 0.1 wt% for the XLPEGDMA or 1 wt% for the XL – p(HEMA), XL – p(HPMA-co-GMA), or XL – p(HEMA-co-GMA) films. The homogonous solutions were then clamped between two quartz glass casting plates that were separated using spacers that effectively controlled the thickness of the cross-linked films and irradiated with 120 $\mu J/cm^2$ of 312 nm light for a specified time (which was either 90 seconds for the XLPEGDMA films or 600 seconds for the XL – p(HEMA), XL – p(HPMA-co-GMA), or XL – p(HEMA-co-GMA) films). For convenience, the synthetic recipes used to create the films used are provided in Table S1. All polymer films were equilibrated in de-ionized water for at least 48 hours before further use, and over this period the de-ionized water solution was changed at least twice to facilitate the equilibration of the polymers with the de-ionized water.

Table S1. Synthetic recipes used to synthesize the films in this study and their resulting properties.

Amount co-monomer in pre-polymerization solution [g/g (vinyl monomer)]			Number of repeat units in PEGDMA, n	$\phi_{s,r}$[a]	Cross-link fraction[b], X	Hydroxyl group content[c]	Water sorption coefficient, K_w	Polymer mesh size, ζ [Å]	Dielectric Constant, ε_m	Salt sorption coefficient, K_s
HEMA	HPMA	GMA								
**Cross-linked poly(ethylene glycol dimethacrylate) (XLPEGDMA)**										
--	--	--	9	0	1	--	0.20 ± 0.01	5.4	11 ± 1	0.07 ± 0.001
--	--	--	9	0.35	1	--	0.33 ± 0.03	7.2	25 ± 5	0.15 ± 0.002
--	--	--	9	0.52	1	--	0.43 ± 0.01	8.9	33 ± 4	0.22 ± 0.01
--	--	--	13	0	1	--	0.43 ± 0.03	9.2	28[d]	0.25 ± 0.02
--	--	--	13	0.36	1	--	0.47 ± 0.01	9.4	31[d]	0.29 ± 0.00
--	--	--	13	0.53	1	--	0.59 ± 0.01	10.5	40[d]	0.51 ± 0.02
**Cross-linked poly(hydroxyethyl methacrylate-co-glycidyl methacrylate) (XL – p(HEMA-co-GMA))**										
0.50	--	0.50	9	0	0.091	3.49	0.20 ± 0.02	8.5	7.5 ± 0.9	0.05 ± 0.001
0.75	--	0.25	9	0	0.091	5.24	0.27 ± 0.03	11.0	12.2 ± 0.7	0.11 ± 0.01
1.00	--	0	9	0	0.091	6.98	0.35 ± 0.02	18.6	15.7 ± 0.4	0.18 ± 0.04
**Cross-linked poly(hydroxyethyl methacrylate) (XL – p(HEMA))**										
1	--	--	9	0	0.091	6.98	0.35 ± 0.02	18.6	15.7 ± 0.4	0.18 ± 0.04
1	--	--	9	0	0.038	7.37	0.37 ± 0.02	20.0	19 ± 1	0.30 ± 0.01
1	--	--	9	0	0.010	7.61	0.40 ± 0.02	26.9	23 ± 3	0.39 ± 0.06
1	--	--	9	0	0	7.68	0.44 ± 0.01	--	34 ± 3	0.39 ± 0.06

Amount co-monomer in pre-polymerization solution [g/g (vinyl monomer)]			Number of repeat units in PEGDMA, n	$\phi_{s,r}$[a]	Cross-link fraction[b], X	Hydroxyl group content[c]	Water sorption coefficient, K_w	Polymer mesh size, ζ [Å]	Dielectric Constant, ε_m	Salt sorption coefficient, K_s
HEMA	HPMA	GMA								
**Cross-linked poly(hydroxypropyl methacrylate-co-glycidyl methacrylate) (XL – p(HPMA-co-GMA))**										
--	0.5	0.5	9	0	0.091	3.15	0.10 ± 0.01	6.9	5.8 ± 0.3	0.018 ± 0.003
--	0.75	0.25	9	0	0.091	4.72	0.17 ± 0.01	8.2	10.2 ± 1.1	0.044 ± 0.004
--	1.0	0	9	0	0.091	6.31	0.22 ± 0.01	9.4	12.3 ± 1.0	0.082 ± 0.004

[a] Volume fraction of solvent in the pre-polymerization mixture. Calculated assuming volume additivity of monomers and water.

[b] Mass fraction of cross-linker in pre-polymerization mixture in units of [mass (cross-linker)/mass (cross-linker and comonomers)]

[c] Units of [mEq./g (dry polymer)]

[d] Polymer films were too fragile to sustain the sample preparation procedure required for the dielectric relaxation spectroscopy measurement, and the dielectric constant was estimated from the water-continuous application of the Maxwell – Garnett model using a dry polymer dielectric constant of 3.12, which is its value for poly(methyl methacrylate)[3]

Section S2. Water/salt sorption measurement

The water/salt sorption selectivity properties of the polymers were determined as the ratio of the water and salt sorption coefficients that were characterized using commonly reported gravimetric weighing and desorption techniques, respectively.[4,5] First, the water sorption properties of the polymers were characterized by weighing the water-equilibrated polymers to determine their mass when equilibrated with de-ionized water, m_{wet}, subsequently drying the polymers under vacuum at 80ºC for at least 48 hours, and weighing the dried polymers to obtain their dry mass, m_{dry}. Using these dry and wet masses, the polymer water uptake, w_u, was calculated as:

$$w_u = \frac{m_{wet} - m_{dry}}{m_{dry}} \quad \text{Eq. S1}$$

Afterwards, the polymers were submerged in an auxiliary solvent that was verified to partition negligibly in the polymers (e.g., cyclohexane), and weighed once more to obtain an auxiliary mass, m_{aux}, which was used to calculate the polymer dry density, ρ_p, as

$$\rho_p = \frac{m_{aux}}{m_{aux} - m_{dry}}(\rho_{aux} - \rho_{air}) \quad \text{Eq. S2}$$

where ρ_{air} and ρ_{aux} are the densities of air and the auxiliary solvent, respectively. The water sorption coefficient, K_w, which was taken as the water volume fraction,[6] was then calculated directly from the water uptake and dry density as:

$$K_w = \frac{w_u}{w_u + \rho_w/\rho_p} \quad \text{Eq. S3}$$

The salt sorption coefficients of the polymers were then determined using a desorption technique. Polymer samples, which were cut into disks, were first equilibrated in 1 M NaCl

solutions for at least 48 hours (these solutions were also changed twice within this time period to facilitate the equilibration of the polymer with the aqueous NaCl solution). The polymers were then blotted dry, and their thickness and diameter were measured using digital calipers. The polymers were then placed in an aliquot of de-ionized water in a jacketed vessel that was controlled at 25 ºC. This de-ionized water was used as a desorption solution to extract the sorbed salt from the polymer matrix, and the conductivity of this solution was measured as a function of time. This conductivity was then used to determine the concentration of salt in the desorption solution using a conductivity/concentration calibration relationship. Using the concentration of the desorption solution, the salt sorption coefficient of the polymer was calculated as:

$$K_s = \frac{C_d V_d}{C_s^s V_p} \quad \text{Eq. S4}$$

where C_d is the concentration of salt in the desorption solution, V_d is the volume of the desorption solution, C_s^s is the concentration of salt that the polymer was initially equilibrated with (i.e., 1 M NaCl), and V_p is the volume of the polymer, which was determined geometrically from the measured thickness and diameter of the film.

The time for the desorption experiment was determined as the time required for 99% of the salt in the polymer to desorb into the external solution as modeled using an application of a classic flat-sheet Fickian desorption model that is commonly used to characterize desorption of a penetrant from a plane-sheet.[7] This model requires information about the polymer thickness, L, which was measured before the experiment, and the salt diffusion coefficient, D_s, which was initially estimated using the one parameter free volume theory and the polymer water volume fraction.[8] Note that generally, the one parameter free volume theory underestimates NaCl diffusion coefficients, so the estimate of the experimental timescale is an overestimate. After the experiment

was complete, the experimental data was fit to the model to verify that the desorption process proceeded to the full extent.

Section S3. Dielectric relaxation spectroscopy measurement

The dielectric constant and state of water properties were characterized using a previously reported two-port dielectric relaxation spectroscopy technique.[9] In this technique, coaxial transmission lines were used to subject a hydrated polymer sample to an oscillating electromagnetic field over a frequency range of 45 MHz to 26.5 GHz that was generated by a vector network analyzer (VNA). The amplitude and phase of the electromagnetic radiation that was transmitted through and reflected from the polymer was measured and interpreted as S-parameters that are mathematically related to the complex relative permittivity of the hydrated polymer sample. A coaxial sample holder was used to connect the polymer sample to the transmission line, and the hydrated polymer film samples were cut into 0.5 cm wide rectangular strips, blotted dry to remove excess surface water, and wrapped around the inner conductor of the sample holder until the polymer filled the annulus of the sample holder.

For the XLPEGDMA polymers, the hydrated films were too fragile to sustain the wrapping procedure used to fill the annulus of the sample holder. As a result, dry XLPEGDMA strips, which were less brittle than the hydrated samples, were wrapped around the inner conductor. The sample holder was then fully submerged in an aliquot of de-ionized water for 48 hours to hydrate the films inside. To remove excess water after this hydration procedure, the interior of the sample holder was thoroughly blotted dry and subject to an air purge for approximately 20 seconds to facilitate removal of additional surface water, and to account for swelling of the polymer films during the

hydration procedure, the sample holder was checked after the measurement to ensure there were no air gaps and the thickness of the sample in the annulus (i.e., the width of the rectangular strip) was measured after the measurement.

The frequency-dependent complex relative permittivity obtained from this measurement was fit to a two-parameter Havrilak-Negami model, as[10]

$$\varepsilon^* = \varepsilon_\infty + \sum_{j=1}^{2} \frac{\Delta\varepsilon_j}{\left[1 + \left(i\omega\tau_j\right)^{1-\alpha_j}\right]^{\beta_j}} \qquad \text{Eq. S5}$$

where $\Delta\varepsilon_j$ and τ_j are the dielectric strength and characteristic timescale, respectively, of the jth relaxation, ε_∞ is the high-frequency static permittivity, ω is the angular frequency, and α_j and β_j, are empirically determined shape parameters (the best fit for the spectra obtained here were obtained using $\alpha_j = \beta_j = 1$, which is a special case of the Havriliak-Nagami model referred to as an ideal Debye relaxation). The model was constrained so that the first relaxation process corresponded to a bulk-like water relaxation (i.e., $\tau_1 = 8.8$ ps),[11] and that the second relaxation corresponded to a restricted water molecule relaxation (i.e., $\tau_2 > 8.8$ ps).

The dielectric constant and concentration of irrotationally bound water molecules (i.e., water in the polymer that cannot contribute to dipolar relaxation processes during the DRS measurement) was extracted from the Havriliak-Nagami parameters. The dielectric constant (i.e., the static relative permittivity) is related to this data because it quantifies the capacity of the hydrated polymer sample to polarize in the presence of an electromagnetic field. The state of water properties of the hydrated polymer can be obtained from the relative permittivity data by calculating the concentration of polarizable water (i.e., that which contributes to the dielectric

strength) in the hydrated polymer and combining this information with the total water concentration in the hydrated polymer.

The dielectric constant is calculated as the sum of the high frequency permittivity and the dielectric strength of all the relaxation processes as:[10]

$$\varepsilon_m = \varepsilon_\infty + \sum_j \Delta\varepsilon_j \quad \text{Eq. S6}$$

The concentration of irrotationally bound water, $c_{w,IB}^{m}$, was determined by combining a mass-balance on the total concentration of water in the polymer, c_w^m

$$c_w^m = c_{w,DRS}^{m} + c_{w,IB}^{m} \quad \text{Eq. S7}$$

where $c_{w,DRS}^{m}$ is the concentration of all the water in the polymer that can contribute to dipolar relaxation processes, with an application of the Kirkwood-Froelich equation that relates the concentration of water in each state to the dielectric strength of the relaxation process corresponding to that state, as[12]

$$c_{w,DRS}^{m,ap} = c_w^s F_{KF} \frac{2\varepsilon_m + \varepsilon_\infty}{\varepsilon_m} \sum_j \Delta\varepsilon_j \quad \text{Eq. S8}$$

where c_w^s is the concentration of water in the external solution. The superscript ap is used to denote that Eq. S8 is calculated by normalizing the Kirkwood – Froehlich Equation written for a specific relaxation process to that of de-ionized water, using the function F_{KF}, which is related to the relative permittivity properties of de-ionized water as[12]

$$F_{KF} = \frac{\varepsilon_s}{(\varepsilon_s - \varepsilon_\infty^s)(2\varepsilon_s + \varepsilon_\infty^s)} \quad \text{Eq. S9}$$

where ε_s and ε_∞^s are the low and high frequency limits of the relative permittivity of de-ionized water.

Section S4. Network mesh size calculation

The network mesh size of the polymers was calculated from the swelling properties (i.e., the water sorption data) using the Peppas – Lucht equation that describes the mesh size of non-Gaussian chains.[13,14] First, the number-average molecular weight between cross-links, $\bar{M}_c$, was calculated using

$$\frac{1}{\bar{M}_c} = \frac{2}{\bar{M}_n} - \frac{\frac{1}{\rho_p V_w}\left[\ln\left(1-\phi_p\right)+\phi_p+\chi\phi_p^2\right]\left[1-\frac{1}{N}\left(\frac{\phi_p}{\phi_{p,r}}\right)^{2/3}\right]}{\phi_{p,r}\left(\left(\frac{\phi_p}{\phi_{p,r}}\right)^{1/3}-\frac{1}{2}\left(\frac{\phi_p}{\phi_{p,r}}\right)\right)\left(1+\frac{1}{N}\left(\frac{\phi_p}{\phi_{p,r}}\right)^{1/3}\right)^2} \quad \text{Eq. S10}$$

where $\bar{M}_n$ is the number average molecular weight of the polymer chains prior to cross-linking, $\phi_{p,r}$ is the volume fraction of polymer in the relaxed state (i.e., before cross-linking such that $\phi_{p,r} = 1 - \phi_{s,r}$), ϕ_p is the polymer volume fraction in the polymer after cross-linking and swollen in water (i.e., $\phi_p = 1 - \phi_w$), V_w is the molar volume of water, and χ is the polymer-solvent interaction parameter (i.e., the so-called Flory-Huggins interaction parameter). N represents the number of links of the polymer chain, which is calculated as[13]

$$N = \lambda \bar{M}_c / M_r \quad \text{Eq. S11}$$

where λ is the polymer backbone bond factor and M_r is the molecular weight of the repeat unit between the cross-links. In the application of Eq. S10 to polymer networks that are prepared under

conditions where the chain growth occurs concurrent with cross-link formation (and because it is impossible to measure $\bar{M}_n$ before cross-linking), it is common to assume that the conversion of the monomers is sufficient such that the term $2/\bar{M}_n$ is small and can be neglected in Eq. S10.[14,15] Once the number average molecular weight between cross-links is determined, the root-mean-square end-to-end distance of the polymer chains can be calculated as[13]

$$(\bar{r}_0^2)^{1/2} = (C_n N l^2)^{1/2} \qquad \text{Eq. S12}$$

where l is the average bond length along the backbone of the repeat unit (i.e., 1.54 Å for C–C bonds, as is relevant for all the materials considered here). Finally, the mesh size is obtained directly using the root-mean-square end-to-end distance as:

$$\zeta = \phi_p^{-\frac{1}{3}}(\bar{r}_0^2)^{1/2} \qquad \text{Eq. S13}$$

For convenience, the parameters used to determine the network mesh size (i.e., Flory's characteristic ratio and the relations used to determine the hydration-dependent Flory-Huggins parameter) are reported in Table S2.

Table S2. Parameters used for the calculation of the polymer mesh size.

Material	Flory-Huggins interaction parameter, χ	Flory's characteristic ratio, C_n
Hydroxyl-containing methacrylates[a]	$\chi = 0.32 + 0.904\phi_p$	7
XLPEGDMA[16,17]	$\chi = 0.28 + 0.892\phi_p$	4

[a]Data obtained for XL – p(HEMA), XL – p(HEMA-co-GMA), and XL – p(HPMA-co-GMA), as reported for HEMA-containing polymers reported by Peppas et al.[15] Note that the relationship for the hydration-dependent Flory-Huggins parameter was obtained from direct regression of the data reported in Table 1 of Peppas et al.[15]

Section S5. Additional figures

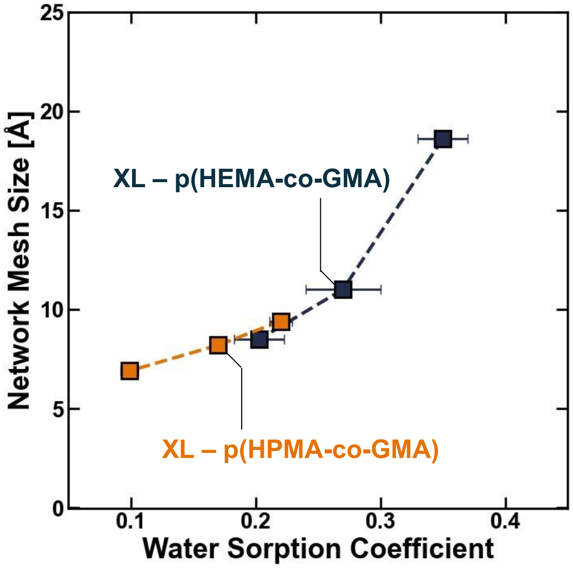

Figure S1. The network mesh size of XL – p(HEMA-co-GMA) (■) and XL – p(HPMA-co-GMA) (■) plotted as a function of water sorption coefficient. The dashed lines are drawn to guide the eye, and the standard deviations are calculated from the mean of three measurements.

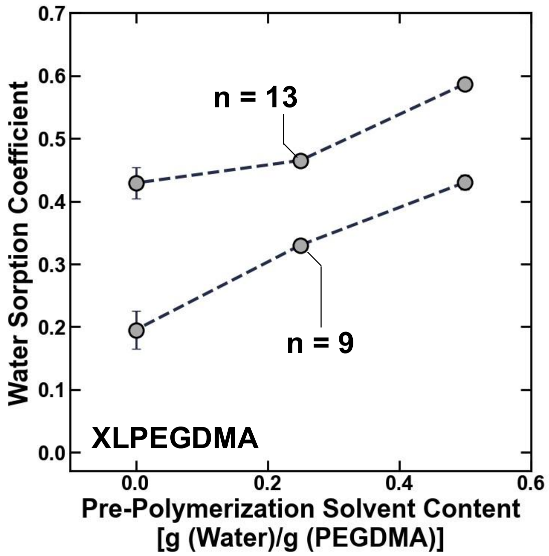

Figure S2. The water sorption coefficient of XLPEGDMA films (n = 9 and n=13) plotted as a function of the water volume fraction of the pre-polymerization mixtures. The dashed lines are drawn to guide the eye, and the standard deviations are calculated from the mean of three measurements.

Section S6. References

(1) Luo, H.; Chang, K.; Bahati, K.; Geise, G. M. Functional Group Configuration Influences Salt Transport in Desalination Membrane Materials. *Journal of Membrane Science* **2019**, *590*, 117295. https://doi.org/10.1016/j.memsci.2019.117295.

(2) Ju, H.; McCloskey, B. D.; Sagle, A. C.; Kusuma, V. A.; Freeman, B. D. Preparation and Characterization of Crosslinked Poly(Ethylene Glycol) Diacrylate Hydrogels as Fouling-Resistant Membrane Coating Materials. *Journal of Membrane Science* **2009**, *330* (1–2), 180–188. https://doi.org/10.1016/j.memsci.2008.12.054.

(3) Geise, G. M. Fundamental Water and Salt Transport Properties of Polymeric Materials. *Progress in Polymer Science* **2014**.

(4) Bannon, S. M.; Kutner, E.; Garretson, B.; Geise, G. M. Understanding the Influence of Sodium Chloride Concentration on Ion Diffusion in Charged Polymers. *Journal of Membrane Science* **2024**, *712*, 123197. https://doi.org/10.1016/j.memsci.2024.123197.

(5) Bannon, S. M.; Geise, G. M. Influence of Donnan and Dielectric Exclusion on Ion Sorption in Sulfonated Polysulfones. *Journal of Membrane Science* **2024**, 122396. https://doi.org/10.1016/j.memsci.2023.122396.

(6) Xie, W.; Cook, J.; Park, H. B.; Freeman, B. D.; Lee, C. H.; McGrath, J. E. Fundamental Salt and Water Transport Properties in Directly Copolymerized Disulfonated Poly(Arylene Ether Sulfone) Random Copolymers. *Polymer* **2011**, *52* (9), 2032–2043. https://doi.org/10.1016/j.polymer.2011.02.006.

(7) Crank, J. *The Mathematics of Diffusion*, 2d ed.; Clarendon Press: Oxford, [Eng], 1975.

(8) Zhang, H.; Geise, G. M. Modeling the Water Permeability and Water/Salt Selectivity Tradeoff in Polymer Membranes. *Journal of Membrane Science* **2016**, *520*, 790–800. https://doi.org/10.1016/j.memsci.2016.08.035.

(9) Chang, K.; Luo, H.; Geise, G. M. Water Content, Relative Permittivity, and Ion Sorption Properties of Polymers for Membrane Desalination. *Journal of Membrane Science* **2019**, *574*, 24–32. https://doi.org/10.1016/j.memsci.2018.12.048.

(10) Woodward, W. H. H. Broadband Dielectric Spectroscopy—A Practical Guide. In *ACS Symposium Series*; Woodward, W. H. H., Ed.; American Chemical Society: Washington, DC, 2021; Vol. 1375, pp 3–59. https://doi.org/10.1021/bk-2021-1375.ch001.

(11) Lu, Z.; Lanagan, M.; Manias, E.; Macdonald, D. D. Two-Port Transmission Line Technique for Dielectric Property Characterization of Polymer Electrolyte Membranes. *J. Phys. Chem. B* **2009**, *113* (41), 13551–13559. https://doi.org/10.1021/jp9057115.

(12) Buchner, R.; Hefter, G. T.; May, P. M. Dielectric Relaxation of Aqueous NaCl Solutions. *J. Phys. Chem. A* **1999**, *103* (1), 1–9. https://doi.org/10.1021/jp982977k.

(13) Richbourg, N. R.; Peppas, N. A. The Swollen Polymer Network Hypothesis: Quantitative Models of Hydrogel Swelling, Stiffness, and Solute Transport. *Progress in Polymer Science* **2020**, *105*, 101243. https://doi.org/10.1016/j.progpolymsci.2020.101243.

(14)Lin, H.; Kai, T.; Freeman, B. D.; Kalakkunnath, S.; Kalika, D. S. The Effect of Cross-Linking on Gas Permeability in Cross-Linked Poly(Ethylene Glycol Diacrylate). *Macromolecules* **2005**, *38* (20), 8381–8393. https://doi.org/10.1021/ma0510136.

(15)Peppas, N. A.; Moynihan, H. J.; Lucht, L. M. The Structure of Highly Crosslinked Poly(2-hydroxyethyl Methacrylate) Hydrogels. *J. Biomed. Mater. Res.* **1985**, *19* (4), 397–411. https://doi.org/10.1002/jbm.820190405.

(16)Jang, E.-S.; Kamcev, J.; Kobayashi, K.; Yan, N.; Sujanani, R.; Dilenschneider, T. J.; Park, H. B.; Paul, D. R.; Freeman, B. D. Influence of Water Content on Alkali Metal Chloride Transport in Cross-Linked Poly(Ethylene Glycol) Diacrylate.1. Ion Sorption. *Polymer* **2019**, *178*, 121554. https://doi.org/10.1016/j.polymer.2019.121554.

(17)Stringer, J. L.; Peppas, N. A. Diffusion of Small Molecular Weight Drugs in Radiation-Crosslinked Poly(Ethylene Oxide) Hydrogels. *Journal of Controlled Release* **1996**, *42* (2), 195–202. https://doi.org/10.1016/0168-3659(96)01457-5.
